# Underdog Expectations and Employees’ Interpersonal Counterproductive Work Behavior: The Mediating Roles of Perceived Insider Status and Moral Disengagement

**DOI:** 10.3390/bs16050799

**Published:** 2026-05-17

**Authors:** Huichi Qian, Jin Cheng, Yuan Yuan, Tao Zhang

**Affiliations:** School of Management, Xiamen University, Xiamen 361000, China; 17620220156356@stu.xmu.edu.cn (H.Q.); chengjin1025@xmu.edu.cn (J.C.); 17620221151243@stu.xmu.edu.cn (T.Z.)

**Keywords:** underdog expectations, interpersonal counterproductive work behavior, moral disengagement, perceived insider status, organization-based self-esteem

## Abstract

As organizational competition intensifies, employees have become increasingly responsive to evaluative cues from their work environment. Among these, underdog expectations—employees’ perceptions that others view them as unlikely to succeed—can trigger strong psychological reactions that shape interpersonal behavior. Drawing on self-determination theory, this study examines how underdog expectations influence employees’ interpersonal counterproductive work behavior (CWB-I). Using a three-wave time-lagged survey design with 221 employees, we found that underdog expectations positively predict CWB-I through two parallel psychological mechanisms: increased moral disengagement and reduced perceived insider status. In addition, organization-based self-esteem (OBSE) strengthens these indirect effects, such that the mediating relationships are stronger among employees with high OBSE. These findings extend research on underdog expectations by revealing both relational and cognitive pathways linking negative evaluative expectations to interpersonal deviance, while also highlighting the complex role of self-evaluative organizational identity in shaping employees’ behavioral responses to status-based threats.

## 1. Introduction

In contemporary organizations characterized by heightened competition and increasing social comparison, employees are frequently exposed to evaluative cues regarding their expected likelihood of success (Mohd et al., 2023; Ng, 2017). Among these cues, underdog expectations refer to individuals’ perceptions that they are seen by others as unlikely to succeed, representing a persistent form of negative social evaluative signaling regarding their expected future performance and competence (Nurmohamed, 2020). Although such expectations may appear as passive judgments, they can undermine employees’ psychological functioning and, in turn, shape their behavioral responses in the workplace (Sun & Yu, 2025).

Self-determination theory (SDT; Ryan & Deci, 2000, 2017) provides a useful framework for understanding how social evaluative cues influence employees’ psychological functioning and behavior. SDT posits that individuals have three basic psychological needs—autonomy, competence, and relatedness—whose satisfaction is essential for optimal functioning, whereas their frustration leads to maladaptive outcomes (Ryan & Deci, 2000, 2017). Within this framework, underdog expectations can be understood as a form of competence-based negative evaluation that undermines employees’ perceived ability and social standing (Y. J. Kim & Kim, 2020; Shapiro & Neuberg, 2007; Teng et al., 2021). By signaling that an individual is unlikely to succeed, such expectations threaten employees’ sense of competence, while simultaneously conveying social devaluation that weakens their sense of relatedness within the organization (Ryan & Deci, 2000, 2017). In addition, such evaluative cues may also undermine employees’ sense of autonomy to some extent by constraining their perceived agency and volitional control in pursuing work-related goals (Ryan & Deci, 2000, 2017). As a result, underdog expectations are likely to induce psychological need frustration, which, in turn, gives rise to various maladaptive psychological and behavioral responses.

Building on self-determination theory, we argue that need frustration induced by underdog expectations operates through two distinct and theoretically separable psychological mechanisms: a relational identity pathway and a cognitive moral regulation pathway. First, from a relatedness perspective, underdog expectations convey persistent signals of social devaluation and interpersonal exclusion, thereby undermining employees’ perceived insider status (Nurmohamed, 2020; Ryan & Deci, 2000, 2017). Perceived insider status reflects the extent to which employees feel accepted, included, and recognized as legitimate members of the organization (Stamper & Masterson, 2002). When employees perceive themselves as outsiders rather than valued insiders, they are more likely to experience relational detachment and reduced psychological attachment to the organization and its members (Caza et al., 2018; Stamper & Masterson, 2002). This relational disconnection subsequently increases the likelihood of interpersonal counterproductive work behavior. This pathway captures a relational identity-based response to need frustration, primarily rooted in relatedness need deprivation. Second, from a competence-based perspective, underdog expectations signal low expectations of success and capability, thereby threatening employees’ sense of competence (Nurmohamed, 2020; Ryan & Deci, 2000, 2017). In response to this competence-related threat, employees may engage in moral disengagement, which refers to cognitive processes that enable individuals to justify or rationalize norm-violating behavior without self-sanction (Lin & Xiao, 2023; Moore et al., 2012). This mechanism allows employees to reinterpret interpersonal harm as acceptable under conditions of perceived devaluation or unfair treatment. This pathway reflects a cognitive moral regulation response, through which competence need frustration translates into weakened moral self-regulation. Taken together, these two pathways are conceptually distinct in both their psychological bases and functional roles: perceived insider status reflects a relational and identity-based evaluation of organizational belonging, whereas moral disengagement reflects a cognitive mechanism of moral justification and self-regulatory disengagement. This dual-pathway logic clarifies how underdog expectations trigger different psychological processes that jointly contribute to interpersonal counterproductive work behavior.

Building on the dual-pathway model, we further propose that the indirect effects of underdog expectations on interpersonal counterproductive work behavior vary as a function of organization-based self-esteem (OBSE). OBSE refers to employees’ global self-evaluation of their competence, value, and worth as organizational members (Pierce et al., 1989), reflecting the extent to which organizational membership is central to one’s self-concept. Because underdog expectations constitute salient negative evaluative signals regarding employees’ organizational value and competence, individuals with higher OBSE are more strongly affected by such context-specific evaluations, as these cues directly threaten a core component of their self-definition (Nurmohamed, 2020; Pierce et al., 1989). Specifically, OBSE intensifies the relational identity pathway by making employees’ sense of organizational belonging more contingent on external evaluative feedback. When high-OBSE employees perceive underdog expectations, the resulting discrepancy between their positive self-view and external devaluation is more pronounced, thereby strengthening the erosion of perceived insider status and subsequent interpersonal withdrawal (J. Yang et al., 2025). Similarly, OBSE strengthens the cognitive moral regulation pathway by increasing the psychological salience of competence-related self-threat. For individuals who strongly define themselves as capable and valuable organizational members, negative performance expectations are more likely to trigger self-defensive cognitive processes (Sun & Yu, 2025), thereby facilitating moral disengagement as a means of resolving self-consistency tension. Taken together, OBSE functions as a self-referential boundary condition that amplifies both relational and cognitive responses to underdog expectations, thereby strengthening the indirect effects on interpersonal counterproductive work behavior.

**Figure 1:**
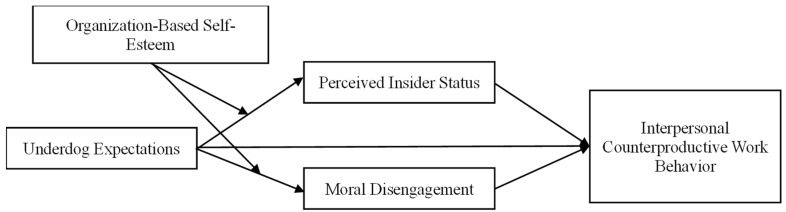
Theoretical Model.

This study proposes a moderated parallel mediation model in which underdog expectations exert their effects on interpersonal counterproductive behavior through perceived insider status and moral disengagement, with OBSE functioning as a dual moderator. This study makes three key theoretical contributions. First, this study extends research on underdog expectations beyond its predominant focus on task-related and individual-level outcomes—such as job performance, work motivation, and unethical behaviors such as cheating (Nurmohamed, 2020; Teng et al., 2021)—to examine its implications for interpersonal counterproductive work behavior (CWB-I). By doing so, we highlight the relationally harmful consequences of negative evaluative expectations in organizations, thereby broadening the scope of underdog expectations research from individual task functioning to interpersonal deviance. Second, by integrating self-determination theory with research on identity threat and moral self-regulation, this study identifies two parallel psychological mechanisms through which underdog expectations influence interpersonal deviance—perceived insider status, reflecting relational identity disruption associated with relatedness need frustration, and moral disengagement, reflecting cognitive moral regulation processes linked to competence-related need threat. Together, these pathways distinguish relational versus cognitive responses to negative evaluative signals. Third, this study offers a counterintuitive insight into the role of organization-based self-esteem (OBSE) as a boundary condition. Contrary to the conventional view that OBSE serves as a buffering psychological resource (Pierce & Gardner, 2004; Bowling et al., 2010), our findings reveal that OBSE amplifies the indirect effects of underdog expectations on interpersonal counterproductive work behavior. This paradox suggests that individuals who strongly identify with their organization may be more vulnerable to negative evaluative cues, thereby challenging prevailing assumptions in the self-concept literature and highlighting the complex role of context-specific self-views in shaping behavioral responses to social evaluation. The theoretical model is illustrated in Figure 1.

## 2. Theory and Hypotheses

### 2.1. The Effect of Underdog Expectations on Interpersonal Counterproductive Work Behavior

Self-determination theory posits that the satisfaction of employees’ basic psychological needs for competence, relatedness, and autonomy is essential for adaptive functioning and constructive workplace behavior, whereas frustration of these needs contributes to maladaptive and norm-violating responses (Ryan & Deci, 2000, 2017).

Building on this perspective, we argue that underdog expectations constitute a form of persistent negative evaluative signaling that threatens employees’ perceived competence and social standing, thereby inducing psychological need frustration. Such expectations communicate low expectations regarding employees’ future success and ability, which may undermine their sense of competence, while simultaneously conveying social devaluation that weakens their perceived relational standing within the organization (Nurmohamed, 2020; Sun & Yu, 2025).

When employees experience sustained exposure to such negative evaluative cues, they are more likely to develop unfavorable interpersonal orientations toward organizational members as a way of coping with perceived devaluation and psychological strain (Teng et al., 2021). This may manifest in interpersonal counterproductive work behavior (CWB-I), defined as intentional behaviors that violate organizational norms and harm other members of the organization (Robinson & Bennett, 1995). Based on this analysis, the study proposes the following hypothesis:

**H1.**  *Underdog expectations positively predict interpersonal counterproductive work behavior.*

### 2.2. The Mediating Role of Perceived Insider Status in the Relationship Between Underdog Expectations and Interpersonal Counterproductive Work Behavior

Underdog expectations convey persistent signals that employees are perceived as unlikely to succeed, which can undermine their sense of acceptance, belonging, and inclusion within the organization (Hakak, 2015). From a self-determination theory perspective, such perceptions primarily frustrate employees’ relatedness need, which reflects the need to feel connected to and valued by others in the workplace (Ryan & Deci, 2000, 2017).

Perceived insider status captures employees’ subjective sense of being a core and valued member of the organization (Knapp et al., 2014; Stamper & Masterson, 2002; Wang & Kim, 2013). When employees are exposed to underdog expectations, the repeated experience of being devalued or socially underestimated weakens their perceived insider status, as they are more likely to feel excluded from the organizational in-group and less recognized as legitimate members (Ashforth et al., 2008; Steele et al., 2002).

A reduced sense of insider status signals a loss of relational connection with the organization and its members, which weakens employees’ willingness to maintain positive interpersonal engagement at work. As employees feel less embedded in the organization, they are more likely to disengage from cooperative norms and become more prone to behaviors that harm interpersonal relationships in the workplace (A. Kim et al., 2019; Liu et al., 2024). Accordingly, reduced perceived insider status increases the likelihood of interpersonal counterproductive work behavior. Based on this analysis, the following hypothesis is proposed:

**H2.**  *Perceived insider status mediates the relationship between underdog expectations and interpersonal counterproductive work behavior.*

### 2.3. The Mediating Role of Moral Disengagement in the Relationship Between Underdog Expectations and Interpersonal Counterproductive Work Behavior

Moral disengagement refers to a cognitive process through which individuals deactivate their internal moral self-regulatory standards, allowing them to engage in unethical or norm-violating behavior without experiencing self-condemnation (Moore et al., 2012). This process involves cognitive restructuring strategies such as moral justification, displacement of responsibility, and dehumanization, which collectively enable individuals to reinterpret harmful behaviors as acceptable under certain conditions (Bandura et al., 1996; Bandura, 1999). Building on self-determination theory, we argue that underdog expectations create persistent competence-related evaluative threats by signaling that employees are perceived as unlikely to succeed or lacking in ability (Nurmohamed, 2020; Ryan & Deci, 2000, 2017). Such repeated competence-based devaluation frustrates employees’ need for competence and generates psychological pressure to cognitively reinterpret negative workplace experiences in a self-protective manner (Ryan & Deci, 2000, 2017; Niven & Healy, 2015).

Under these conditions, employees may become more likely to engage in moral disengagement as a cognitive or moral justification strategy. Specifically, employees exposed to chronic underdog expectations may reinterpret interpersonal mistreatment or harmful conduct as understandable or justified reactions to perceived unfair evaluation and social devaluation (Nurmohamed, 2020; Bandura et al., 1996; Bandura, 1999). Through moral justification, harmful behaviors toward others are reframed as acceptable responses to persistent underestimation (Moore, 2008). At the same time, responsibility for such behaviors may be displaced onto supervisors, coworkers, or organizational evaluation systems, thereby reducing personal accountability for interpersonal harm (Moore, 2008).

Once moral self-regulatory standards are cognitively disengaged, employees are less constrained by internal self-sanctions that would normally inhibit harmful interpersonal behavior. As feelings of guilt and moral accountability are weakened, employees become more willing to engage in interpersonal counterproductive work behavior (CWB-I), including hostility, exclusion, social undermining, and other forms of interpersonal deviance (Detert et al., 2008). Based on the above, we propose the following hypothesis:

**H3.**  *Moral disengagement mediates the relationship between underdog expectations and interpersonal counterproductive work behavior.*

### 2.4. The Moderating Role of Organization-Based Self-Esteem in the Relationship Between Underdog Expectations and Perceived Insider Status

Organization-based self-esteem (OBSE) refers to employees’ overall self-evaluation of their value, competence, and worth as organizational members (Pierce et al., 1989). Unlike perceived insider status, which reflects employees’ sense of belonging and inclusion within the organization, OBSE captures the extent to which individuals define themselves as capable and valuable organizational members (Pierce et al., 1989; Stamper & Masterson, 2002). As a context-specific form of self-esteem, OBSE shapes how strongly employees interpret and react to organizational evaluative cues.

Building on self-determination theory and self-consistency perspectives, we argue that OBSE strengthens the negative relationship between underdog expectations and perceived insider status. Employees with high OBSE typically maintain a strong positive self-view regarding their organizational value and competence (Pierce et al., 1989). Because organizational membership constitutes an important component of their self-definition, negative evaluative signals embedded in underdog expectations become particularly self-relevant and psychologically threatening (Nurmohamed, 2020).

When high-OBSE employees perceive that others view them as unlikely to succeed, the discrepancy between their positive organizational self-evaluation and external devaluation becomes more salient (Betz, 1994; Nurmohamed, 2020; Pierce et al., 1989). As a result, they are more likely to interpret underdog expectations as signals of relational rejection and diminished standing within the organization (Ashforth et al., 2008; Hakak, 2015; Steele et al., 2002). This intensified perception of social devaluation subsequently leads to a sharper decline in perceived insider status. In contrast, employees with low OBSE possess weaker organizational self-evaluations and are therefore less psychologically invested in maintaining a positive organizational self-image. Consequently, underdog expectations may be perceived as less self-threatening, resulting in a comparatively weaker negative effect on perceived insider status. Based on the above analysis, the following hypothesis is proposed:

**H4.**  *Organization-based self-esteem moderates the relationship between underdog expectations and perceived insider status, such that the higher the level of organization-based self-esteem, the stronger the negative effect of underdog expectations on perceived insider status.*

### 2.5. The Moderating Role of Organization-Based Self-Esteem in the Relationship Between Underdog Expectations and Moral Disengagement

Organization-based self-esteem (OBSE) reflects employees’ perceptions of their own value, competence, and worth within the organizational context (Pierce et al., 1989). Employees with high OBSE tend to possess a strong positive self-view regarding their organizational competence and significance. As a result, negative evaluative cues in the workplace become particularly self-relevant and psychologically consequential for these individuals.

Building on self-determination theory and self-consistency perspectives, we argue that OBSE strengthens the positive relationship between underdog expectations and moral disengagement. Underdog expectations communicate that employees are viewed as unlikely to succeed, thereby threatening employees’ competence-based self-evaluations (Nurmohamed, 2020; Ryan & Deci, 2000, 2017). For employees with high OBSE, such external devaluation creates a stronger discrepancy between their positive organizational self-concept and the negative expectations imposed by others (Nurmohamed, 2020; Pierce et al., 1989). To reduce the psychological discomfort associated with this self-consistency threat, high-OBSE employees may become more likely to engage in moral disengagement as a self-defensive cognitive regulation strategy (Kish-Gephart et al., 2014). Specifically, moral disengagement enables individuals to cognitively reinterpret harmful or norm-violating behavior as justified, understandable, or externally caused, thereby protecting their threatened self-concept from further psychological damage (Moore et al., 2012; Moore, 2008; Niven & Healy, 2015; Shalvi et al., 2011). In contrast, employees with low OBSE possess comparatively weaker organizational self-evaluations and are therefore less psychologically invested in maintaining a highly positive organizational self-image (Pierce et al., 1989). Consequently, underdog expectations may generate less self-threatening discrepancy for these individuals, resulting in a weaker tendency toward moral disengagement. Based on the above analysis, the following hypothesis is proposed:

**H5.**  *Organization-based self-esteem moderates the relationship between underdog expectations and moral disengagement, such that the higher the level of organization-based self-esteem, the stronger the positive effect of underdog expectations on moral disengagement.*

### 2.6. The Moderating Role of Organization-Based Self-Esteem in the Indirect Effect of Underdog Expectations on Interpersonal Counterproductive Work Behavior via Perceived Insider Status

The indirect effect of underdog expectations on interpersonal counterproductive work behavior through perceived insider status is expected to vary depending on employees’ levels of organization-based self-esteem (OBSE). For employees with high OBSE, organizational evaluations are more central to their self-concept and perceptions of self-worth (Riketta, 2005). As a result, underdog expectations are more likely to be interpreted as strong signals of relational devaluation and diminished organizational acceptance (Bowling et al., 2010). Consequently, high-OBSE employees experience a sharper decline in perceived insider status when exposed to underdog expectations. This stronger erosion of perceived insider status subsequently increases the likelihood of interpersonal counterproductive work behavior.

In contrast, employees with low OBSE are less psychologically invested in maintaining a highly positive organizational self-evaluation. Therefore, underdog expectations are less likely to generate strong perceptions of relational devaluation, resulting in a comparatively weaker reduction in perceived insider status and a weaker indirect effect on interpersonal counterproductive work behavior. Based on the above analysis, the following hypothesis is proposed:

**H6.**  *Organization-based self-esteem moderates the indirect effect of underdog expectations on interpersonal counterproductive work behavior via perceived insider status, such that the indirect effect is stronger when organization-based self-esteem is high.*

### 2.7. The Moderating Role of Organization-Based Self-Esteem in the Indirect Effect of Underdog Expectations on Interpersonal Counterproductive Work Behavior via Moral Disengagement

The indirect effect of underdog expectations on interpersonal counterproductive work behavior through moral disengagement is expected to vary depending on employees’ levels of organization-based self-esteem (OBSE). For employees with high OBSE, organizational competence and personal value constitute important components of their self-concept (Pierce et al., 1989). Consequently, underdog expectations represent highly self-relevant negative evaluative signals that strongly threaten their competence-based self-evaluations (Collins & Strelan, 2021). When high-OBSE employees perceive that others view them as unlikely to succeed, the discrepancy between their positive organizational self-concept and external devaluation becomes more psychologically salient (Collins & Strelan, 2021). To reduce the discomfort associated with this self-consistency threat, these employees are more likely to engage in moral disengagement as a self-defensive cognitive regulation strategy. Through moral disengagement, harmful or norm-violating interpersonal behaviors can be cognitively reframed as understandable or justified responses to persistent underestimation and devaluation (Moore, 2008). As moral self-regulatory constraints weaken, employees become more willing to engage in interpersonal counterproductive work behavior.

In contrast, employees with low OBSE possess comparatively weaker organizational self-evaluations and are therefore less psychologically invested in maintaining a highly positive organizational self-image. As a result, underdog expectations generate weaker competence-related self-threat, leading to lower levels of moral disengagement and a comparatively weaker indirect effect on interpersonal counterproductive work behavior. Based on this analysis, the following hypothesis is proposed:

**H7.**  *Organization-based self-esteem moderates the indirect effect of underdog expectations on interpersonal counterproductive work behavior via moral disengagement, such that the indirect effect is stronger when organization-based self-esteem is high.*

## 3. Method

### 3.1. Participants

We collected data from employees working in six manufacturing enterprises located in the Yangtze River Delta region of China, a highly developed and competitive economic area. Employees were recruited through department heads, who assisted in distributing the survey link within their respective organizations. Participation was voluntary, and informed consent was obtained from all respondents prior to data collection. No financial or material incentives were provided. A total of 221 valid responses were retained after excluding incomplete or unmatched questionnaires, resulting in an effective response rate of 73.67%.

The final sample consisted of 61.10% male and 38.90% female respondents. Regarding age, 22.60% were 25 years old or younger, 43.40% were between 26 and 35, 22.60% were between 36 and 45, and 11.30% were 46 years or older. In terms of education, 62.00% had a secondary vocational education or below, 23.50% held an associate degree, 14.00% held a bachelor’s degree, and 0.50% held a master’s degree or above. Organizational tenure distribution was as follows: 27.60% had worked in the organization for less than one year, 30.80% for one to three years, 12.70% for three to five years, and 29.00% for more than five years.

Importantly, respondents were drawn from multiple departments and job positions across different firms rather than from intact teams. Employees did not share common supervisors or belong to formally defined work units. Therefore, the data reflect an individual-level structure, and no multilevel clustering was required in the analysis.

### 3.2. Procedure

A three-wave time-lagged survey design was adopted to reduce common method bias. Data collection was conducted at three time points with 10-day intervals between each wave. Respondents were matched across waves using a unique identifier combining the initials of their names and the last four digits of their mobile phone numbers.

At Time 1, demographic information (gender, age, education, and organizational tenure) and underdog expectations were collected. At Time 2, perceived insider status, moral disengagement, and organization-based self-esteem were measured. At Time 3, interpersonal counterproductive work behavior was assessed.

To reduce common method bias, several procedural remedies were implemented following Podsakoff et al. (2003). First, predictor, mediator, moderator, and outcome variables were collected across three separate waves with temporal separation. Second, participants were assured that their responses would remain anonymous and confidential and would be used solely for academic research purposes. Third, participation was voluntary, and respondents were informed that there were no right or wrong answers, thereby reducing evaluation apprehension and socially desirable responding.

The study was conducted in accordance with the Declaration of Helsinki, and approved by the Institutional Review Board of Xiamen University. Informed consent was obtained from all subjects involved in the study.

### 3.3. Measures

All focal constructs were measured using previously validated English-language scales that have been widely used in organizational behavior research. Following Brislin’s (1986) back-translation procedure, all items were translated into Chinese and then back-translated into English to ensure semantic equivalence. All constructs were operationalized as unidimensional measures using a five-point Likert scale ranging from 1 (strongly disagree) to 5 (strongly agree).

Confirmatory factor analysis supported the distinctiveness of the study variables. All constructs demonstrated acceptable reliability and validity, with Cronbach’s alpha and composite reliability (CR) values exceeding the recommended threshold of 0.70. Although the average variance extracted (AVE) value for perceived insider status was slightly below the conventional cutoff of 0.50, its CR remained above 0.70, indicating acceptable convergent validity (Fornell & Larcker, 1981).

Underdog expectations were measured using the three-item scale developed by Nurmohamed (2020). An example item is “I am viewed as an underdog in doing this job by other individuals”. The Cronbach’s alpha was 0.75.

Perceived insider status was measured using the six-item scale developed by Stamper and Masterson (2002). An example item is “I feel very much a part of my work organization”. The Cronbach’s alpha was 0.72.

Moral disengagement was measured using the eight-item scale developed by Moore et al. (2012). An example item is “It is okay to spread rumors to defend those you care about”. The Cronbach’s alpha was 0.91.

Organization-based self-esteem was measured using the ten-item scale developed by Pierce et al. (1989). An example item is “I count around here”. The Cronbach’s alpha was 0.91.

Interpersonal counterproductive work behavior was measured using the six-item scale developed by Dalal et al. (2009). An example item is “Behaved in an unpleasant manner toward my supervisor/a coworker”. The Cronbach’s alpha was 0.89.

Based on previous research, we included demographic variables such as gender, age, education, and organizational tenure as control variables (Nurmohamed, 2020; Teng et al., 2021).

### 3.4. Data Analysis

Data analysis was conducted using SPSS 22.0, Mplus 8.3, and SmartPLS 4.1.1.8. Descriptive statistics and correlations were first computed using SPSS 22.0. Confirmatory factor analysis (CFA) was conducted in Mplus 8.3 to assess construct validity. Discriminant validity was further examined using the Fornell–Larcker criterion and heterotrait–monotrait (HTMT) ratios in SmartPLS 4.1.1.8.

Prior to hypothesis testing, we assessed key statistical assumptions, including normality, multicollinearity, linearity, homoscedasticity, and independence of observations. Hierarchical regression analyses were performed in SPSS 22.0 to test the hypotheses. Mediation and moderated mediation effects were examined using bootstrapping procedures with 5000 resamples based on Hayes’ process approach. All models included control variables.

## 4. Results

### 4.1. Preliminary Analyses

Prior to hypothesis testing, the assumptions underlying parametric analyses were examined. Skewness and kurtosis values indicated that all variables were within acceptable ranges, suggesting approximate normality. In addition, visual inspection of scatterplots indicated that the relationships among the key variables were approximately linear. The assumption of homoscedasticity was found to be acceptable based on inspection of residual plots. Moreover, given that data were collected from individual respondents across different departments and job positions, the independence of observations was ensured. These results supported the appropriateness of Pearson correlation and regression analyses. The results of the descriptive statistics and correlation analysis are presented in Table 1.

### 4.2. Common Method Bias

Given that all focal variables were collected through employee self-reports, several statistical remedies were adopted to minimize the potential influence of common method bias (Podsakoff et al., 2003). First, Harman’s single-factor test was conducted. The results showed that the first unrotated factor accounted for 30.45% of the total variance, which was below the recommended threshold of 50.00%, suggesting that common method bias was unlikely to be a serious concern. Second, confirmatory factor analyses indicated that the hypothesized five-factor model demonstrated a significantly better fit than the single-factor model (see Table 2), providing additional evidence against substantial common method variance. Finally, to further assess discriminant validity and potential common method concerns, the heterotrait–monotrait ratio (HTMT) and the Fornell–Larcker criterion were examined. All HTMT values ranged from 0.22 to 0.78, below the recommended threshold of 0.85 (Henseler et al., 2015). In addition, the square roots of the average variance extracted (AVE) for all constructs exceeded the corresponding inter-construct correlations, supporting adequate discriminant validity. Collectively, these results suggest that common method bias was unlikely to threaten the validity of the findings.

### 4.3. Descriptive Statistics

Table 1 presents the descriptive statistics and correlations among the study variables. Underdog expectations were positively associated with interpersonal counterproductive work behavior, indicating a weak but significant relationship. Underdog expectations were negatively related to perceived insider status, whereas perceived insider status showed a moderate negative association with interpersonal counterproductive work behavior. In addition, underdog expectations were positively associated with moral disengagement, which in turn was positively related to interpersonal counterproductive work behavior. Overall, the correlation pattern among variables was consistent with the proposed theoretical framework.

### 4.4. Confirmatory Factor Analysis

We employed confirmatory factor analysis (CFA) to validate the discriminant validity of five constructs. Given the large number of items for perceived insider status, moral disengagement, interpersonal counterproductive work behavior, and organization-based self-esteem, and given that these four scales are unidimensional, we utilized a parceling method to handle the items. We applied the factorial algorithm, specifically item-to-construct balance, to process the items for the five constructs (Little et al., 2002; Rogers & Schmitt, 2004). Each construct was processed into three items: arranging items from highest to lowest according to their factor loadings, and then arranging three items per row in descending order, alternating the order from high to low and vice versa. Finally, items in each column were parceled into one item, and the average score was taken as the new item score for CFA (Kishton & Widaman, 1994; C. M. Yang et al., 2010; Teng et al., 2021). The results of the CFA are presented in Table 2. The results indicated that the five-factor model provided the best fit to the data (χ^2^ = 182.59, *df* = 80, χ^2^/*df* = 2.28, CFI = 0.95, TLI = 0.93, RMSEA = 0.08, SRMR = 0.06), suggesting good discriminant validity among the five constructs.

### 4.5. Hypotheses Testing

Prior to hypothesis testing, we assessed potential multicollinearity issues by examining variance inflation factors (VIFs) for all predictor constructs in the structural model. The results indicated that all VIF values ranged from 1.11 to 2.43, which were below the recommended threshold of 5 (Kock, 2017). This suggests that multicollinearity was not a concern in the present study, and that the structural estimates are unlikely to be biased by redundant explanatory variables.

We employed linear regression to test the hypotheses, with the results presented in Table 3. Hypothesis 1 posits that underdog expectations have a positive influence on interpersonal counterproductive work behavior. Model 10 supports Hypothesis 1 (β = 0.20, *p* < 0.01).

Hypothesis 2 posits that perceived insider status mediates the relationship between underdog expectations and interpersonal counterproductive work behavior. The results from Model 2 indicate a negative correlation between underdog expectations and perceived insider status (β = −0.23, *p* < 0.01), and the results from Model 11 show a negative correlation between perceived insider status and interpersonal counterproductive work behavior (β = −0.43, *p* < 0.001). Furthermore, the results from Models 10 and 13 support the mediating role of perceived insider status: Model 10 showed that underdog expectations had a significant positive effect on interpersonal counterproductive work behavior (β = 0.20, *p* < 0.01); however, after adding perceived insider status in Model 13, the direct effect of underdog expectations became non-significant (β = 0.11, n.s.), while perceived insider status was negatively associated with interpersonal counterproductive work behavior (β = −0.41, *p* < 0.001). In addition, we tested the mediating effect by conducting a 5000-times bootstrap re-sampling method using PROCESS, indicating that underdog expectations have a positive effect on interpersonal counterproductive work behavior through perceived insider status (indirect effect = 0.14, 95% CI [0.07, 0.22], excluding 0). Therefore, Hypothesis 2 is supported.

Hypothesis 3 posits that moral disengagement mediates the relationship between underdog expectations and interpersonal counterproductive work behavior. The results from Model 6 indicate a positive correlation between underdog expectations and moral disengagement (β = 0.32, *p* < 0.001). Model 12 shows that moral disengagement is positively correlated with interpersonal counterproductive work behavior (β = 0.53, *p* < 0.001). Furthermore, the results from Models 10 and 14 support the mediating role of moral disengagement: after adding moral disengagement in Model 14, the direct effect of underdog expectations became non-significant (β = 0.04, n.s.), while moral disengagement was positively associated with interpersonal counterproductive work behavior (β = 0.51, *p* < 0.001). Additionally, we tested the mediating effect by conducting a 5000-times bootstrap re-sampling method using PROCESS, which indicated that underdog expectations have a positive effect on interpersonal counterproductive work behavior through moral disengagement (indirect effect = 0.08, 95% CI [0.02, 0.14], excluding 0). Therefore, Hypothesis 3 is supported.

**Figure 2:**
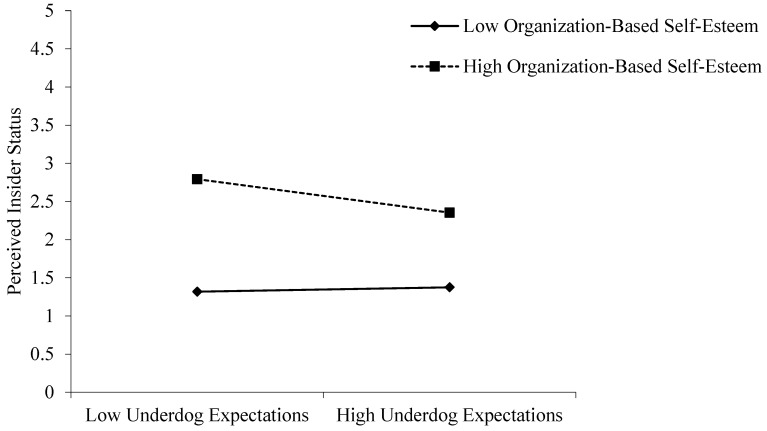
Moderating Role of Organization-Based Self-Esteem in the Relationship between Underdog Expectations and Perceived Insider Status.

Hypothesis 4 posits that organization-based self-esteem strengthens the impact of underdog expectations on perceived insider status. Models 2, 3, and 4 show that the coefficient of interaction was significant (β = −0.12, *p* < 0.05), supporting Hypothesis 4. To further examine the moderating effect of organization-based self-esteem on the relationship between underdog expectations and perceived insider status, the interaction effect was plotted, and simple slope tests were conducted (Aiken & West, 1991). As shown in Figure 2, under higher organization-based self-esteem (M + 1SD), the relationship between underdog expectations and perceived insider status is significant (simple slope = −0.15, *p* < 0.01), while under lower organization-based self-esteem (M−1SD), the relationship between underdog expectations and perceived insider status is not significant (simple slope = −0.02, n.s.). Therefore, Hypothesis 4 is supported.

**Figure 3:**
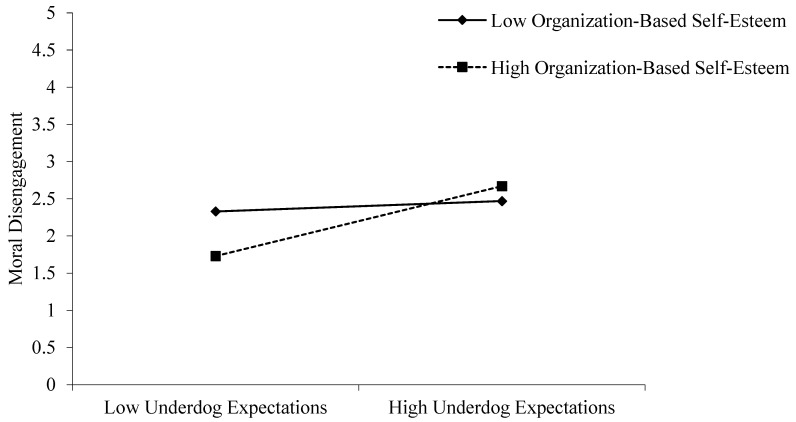
Moderating Role of Organization-Based Self-Esteem in the Relationship between Underdog Expectations and Moral Disengagement.

Hypothesis 5 posits that organization-based self-esteem positively moderates the positive effect of underdog expectations on moral disengagement. Models 6, 7, and 8 show that the interaction coefficient is significant (β = 0.20, *p* < 0.01), supporting Hypothesis 5. To further examine the moderating effect of organization-based self-esteem on the relationship between underdog expectations and moral disengagement, the interaction effect was plotted, and simple slope tests were conducted (Aiken & West, 1991). As shown in Figure 3, under higher organization-based self-esteem (M + 1SD), the relationship between underdog expectations and moral disengagement is significant (simple slope = 0.43, *p* < 0.001), while under lower organization-based self-esteem (M−1SD), the relationship remains significant, but weaker (simple slope = 0.14, *p* < 0.05). Therefore, Hypothesis 5 is supported.

Hypothesis 6 posits that organization-based self-esteem moderates the indirect relationship between underdog expectations and interpersonal counterproductive work behavior through perceived insider status, with the negative relationship being stronger under higher organization-based self-esteem. This study tested the moderated mediation effect by conducting a 5000-times bootstrap re-sampling method using PROCESS. The index of moderated mediation was significant (index = 0.062, 95% CI [0.01, 0.12], excluding 0), indicating that the indirect effect significantly varied across levels of organization-based self-esteem. Specifically, under higher organization-based self-esteem, the indirect effect was significant (indirect effect = 0.08, 95% CI [0.02, 0.13], excluding 0), and under lower organization-based self-esteem, the indirect effect was not significant (indirect effect = 0.00, 95% CI [−0.05, 0.06], including 0). Hypothesis 6 is supported.

Hypothesis 7 posits that organization-based self-esteem moderates the indirect relationship between underdog expectations and interpersonal counterproductive work behavior through moral disengagement, with the positive relationship being stronger under higher organization-based self-esteem. We used the same method as in Hypothesis 6 to test Hypothesis 7, and the results showed that the index of moderated mediation was significant (index = 0.13, 95% CI [0.04, 0.22], excluding 0), indicating that the indirect effect significantly varied across levels of organization-based self-esteem. Specifically, under higher organization-based self-esteem, the indirect effect was significant (indirect effect = 0.20, 95% CI [0.12, 0.30], excluding 0), while under lower organization-based self-esteem, the indirect effect was not significant (indirect effect = 0.05, 95% CI [−0.03, 0.13], including 0). Hypothesis 7 is supported.

## 5. Discussion

This study advances understanding of the interpersonal consequences of underdog expectations by examining how negative social evaluative signals translate into interpersonal counterproductive work behavior (CWB-I). Drawing on self-determination theory, our findings suggest that underdog expectations undermine employees’ psychological functioning and increase interpersonal deviance through two distinct pathways—a relational identity pathway reflected in reduced perceived insider status, and a cognitive moral regulation pathway reflected in heightened moral disengagement. Furthermore, the results reveal that organization-based self-esteem (OBSE) amplifies rather than buffers these indirect effects, suggesting that employees who strongly value their organizational competence and membership may be more psychologically vulnerable to negative evaluative expectations. Together, these findings highlight the important role of psychological need frustration and self-referential evaluation processes in shaping employees’ maladaptive interpersonal responses to underdog expectations.

### 5.1. Theoretical Implications

First, this study extends the literature on underdog expectations by shifting attention from task-related outcomes to interpersonal workplace deviance. Existing research has primarily examined how underdog expectations influence employees’ job performance, work motivation, and individual unethical behaviors such as cheating (Nurmohamed, 2020; Teng et al., 2021). While these studies have established the performance-related consequences of being negatively evaluated, they have paid comparatively limited attention to the broader relational costs of such evaluative experiences. Our findings demonstrate that underdog expectations not only impair employees’ task functioning, but also undermine interpersonal relationships within organizations by increasing interpersonal counterproductive work behavior (CWB-I). Unlike passive withdrawal or individual-focused misconduct, CWB-I directly damages coworkers and supervisors through behaviors such as interpersonal hostility, exclusion, and social undermining (Robinson & Bennett, 1995). By identifying low interpersonal expectations as an antecedent of interpersonal deviance, this study broadens the explanatory scope of underdog expectations research and highlights the relationally destructive consequences of negative workplace evaluations.

Second, this study advances self-determination theory (SDT) by demonstrating how negative social evaluative experiences frustrate employees’ basic psychological needs and subsequently trigger differentiated maladaptive responses. Although prior SDT research has primarily focused on the role of need satisfaction in promoting motivation, well-being, and positive functioning (Ryan & Deci, 2000, 2017), comparatively less attention has been devoted to how workplace evaluative stigma and interpersonal devaluation operate as need-frustrating conditions that shape harmful interpersonal behaviors. Our findings suggest that underdog expectations constitute a psychologically threatening evaluative context that undermines employees’ needs for competence and relatedness, thereby activating two distinct psychological pathways. Specifically, relatedness need frustration manifests through reduced perceived insider status, reflecting employees’ weakened sense of organizational belonging and relational attachment. In contrast, competence-related threat promotes moral disengagement, a self-defensive cognitive regulation mechanism through which employees rationalize norm-violating interpersonal behavior. By distinguishing between a relational identity pathway and a cognitive moral regulation pathway, this study extends SDT beyond its traditional emphasis on positive motivation and adaptive functioning, and demonstrates how differentiated forms of psychological need frustration can produce distinct forms of maladaptive workplace behavior.

Third, this study contributes to the literature on organization-based self-esteem (OBSE) by revealing its counterintuitive amplifying role under conditions of negative social evaluation. Prior research has predominantly conceptualized OBSE as a beneficial psychological resource that buffers employees against workplace stressors and adverse experiences (Bowling et al., 2010; Neves et al., 2020). However, our findings challenge this assumption by demonstrating that high OBSE intensifies, rather than weakens, the negative effects of underdog expectations. Specifically, employees with high OBSE experienced stronger reductions in perceived insider status and greater moral disengagement when exposed to underdog expectations, thereby strengthening the indirect effects on interpersonal counterproductive work behavior. This finding suggests that employees who strongly define themselves as competent and valuable organizational members may become more psychologically vulnerable when confronted with evaluations that threaten this self-view. Accordingly, our study advances the OBSE literature by identifying an important boundary condition under which high organization-based self-esteem functions not as a protective resource, but as a source of heightened sensitivity to self-relevant evaluative threat.

### 5.2. Practical Implications

Our findings suggest that organizations should move beyond generalized employee support strategies and adopt targeted interventions that prevent the formation of underdog expectations, strengthen employees’ organizational belonging, reduce moral disengagement, and provide differentiated management for employees with varying levels of organization-based self-esteem (OBSE). By identifying both the relational identity pathway and the cognitive moral regulation pathway through which underdog expectations translate into interpersonal counterproductive work behavior (CWB-I), this study offers a multi-layered intervention framework for organizational practice.

First, organizations should prioritize preventing the formation of underdog expectations at their source. Because underdog expectations originate from persistent negative evaluative signals regarding employees’ future success and competence, organizations should reduce reliance on subjective, deficit-based judgments in performance evaluation and talent management systems. Instead, supervisors are encouraged to base evaluations and developmental feedback on observable behaviors, objective performance indicators, and behaviorally anchored competency standards. During performance discussions, managers should avoid labeling employees as “unlikely to succeed” or implicitly framing them as organizational “underdogs,” as such deficit-focused evaluations may unintentionally trigger psychological need frustration and subsequent dysfunctional responses. To further reduce the emergence of underdog expectations, organizations can provide supervisor training focused on low-expectation bias awareness, helping managers recognize how implicit assumptions regarding employees’ background, prior performance, or perceived disadvantages may evolve into harmful evaluative expectations. In addition, organizations can establish more transparent and inclusive promotion and development systems to reduce employees’ perceptions of arbitrary or stigmatizing evaluations.

Second, organizations should strengthen employees’ organizational belonging and inclusion in order to disrupt the relational identity pathway linking underdog expectations to interpersonal deviance. Because underdog expectations erode perceived insider status by conveying signals of exclusion and devaluation, organizations should proactively cultivate climates characterized by inclusion, interpersonal respect, and psychological safety. Beyond general climate management, organizations may incorporate targeted assessments of employees’ perceived belonging, experiences of exclusion, and perceptions of being underestimated within regular team climate surveys or organizational feedback systems. Such monitoring mechanisms may help identify teams or supervisory contexts in which underdog expectations are likely to emerge and undermine employees’ perceived insider status. Organizations may also establish low-risk communication channels, such as confidential HR consultations or anonymous feedback systems, through which employees can express concerns regarding experiences of marginalization or devaluation. These interventions directly address employees’ relatedness need frustration and help preserve their sense of organizational membership and social inclusion.

Third, organizations should implement interventions aimed at reducing moral disengagement and cognitive rationalization processes among employees exposed to underdog expectations. Our findings suggest that underdog expectations may weaken employees’ moral self-regulation by fostering defensive cognitive justifications for harmful interpersonal behavior. Accordingly, organizations should move beyond compliance-oriented ethics training and explicitly address the psychological rationalization processes that may arise under conditions of interpersonal devaluation. Team discussions and ethics-related programs can emphasize how feelings of unfairness, exclusion, or being underestimated may encourage employees to justify hostility, noncooperation, or interpersonal undermining toward coworkers. Supervisors should also be trained to identify early indicators of moral disengagement within teams, such as employees normalizing retaliatory behavior or minimizing the consequences of interpersonal harm. Importantly, managerial interventions should not focus solely on punishing deviant behavior after it occurs, but should also address the underlying experiences of devaluation and psychological need frustration that contribute to moral disengagement in the first place. Such practices can help weaken the cognitive moral regulation pathway identified in this study.

Finally, our findings highlight the importance of differentiated management strategies for employees with high organization-based self-esteem (OBSE). Contrary to the traditional assumption that high OBSE universally functions as a protective psychological resource, our results suggest that employees with high OBSE may be particularly vulnerable to negative evaluative cues because organizational competence and membership constitute central components of their self-concept. As a result, underdog expectations may generate stronger identity threat and psychological dissonance among these employees, thereby intensifying declines in perceived insider status and increasing moral disengagement. Managers should therefore recognize that highly confident and organization-identified employees are not necessarily resilient to devaluing feedback. For employees with high OBSE, performance and developmental conversations should emphasize collaborative goal-setting, procedural fairness, and clear behavioral guidance, while avoiding broad negative judgments regarding employees’ long-term potential or organizational value. Providing transparent advancement opportunities and maintaining respectful, competence-affirming communication may help reduce the amplified negative reactions that high-OBSE employees experience when confronted with underdog expectations.

### 5.3. Limitations and Future Research Directions

This study adopted a three-wave time-lagged design to reduce common method concerns; however, several methodological limitations should still be acknowledged. Although temporal separation has been recognized as an effective procedural remedy for mitigating common method bias (Podsakoff et al., 2003), the present design does not fully establish causal relationships among the focal variables. Compared with a single-wave survey design, the multi-wave approach reduced respondents’ ability to infer the full conceptual relationships among variables, thereby alleviating consistency motifs and immediate same-source response bias. Moreover, although the 10-day interval may not fully capture long-term psychological change processes, it introduced temporal separation between predictor, mediator, moderator, and outcome variables, which strengthens internal validity relative to cross-sectional designs. In addition, because the data were collected from a single sample, the confirmatory factor analysis was not cross-validated using an independent sample, which may limit the generalizability and robustness of the measurement model. Future research is encouraged to adopt longer-term longitudinal or quasi-experimental designs, as well as split-sample or multi-group cross-validation approaches, to further enhance causal inference and measurement robustness.

It is important to note that the findings should be interpreted within the cultural context of China, where collectivistic values and high-power distance are prevalent. In such contexts, social evaluation and hierarchical sensitivity may amplify the psychological impact of underdog expectations. Therefore, the observed effects may be contingent upon cultural norms that emphasize relational comparison and authority structures. Future research should examine whether these effects generalize to more individualistic or low power-distance cultures.

Future research may further examine how broader organizational contexts shape employees’ responses to underdog expectations. In increasingly competitive workplaces characterized by ongoing organizational change and heightened performance demands, employees may experience greater pressure to continuously update their knowledge and translate new competencies into effective job performance. Such contextual pressures may increase employees’ sensitivity to evaluative signals regarding their competence and future potential, thereby strengthening the psychological and behavioral consequences of underdog expectations. Future studies may also explore whether organizational factors such as leadership styles, technological change, or AI-enabled work systems amplify or mitigate these effects. For example, toxic leadership may reinforce negative evaluative climates, whereas supportive leadership styles such as servant or transformational leadership may help buffer the harmful interpersonal consequences of underdog expectations (Tavanti, 2011; Eva et al., 2019).

## 6. Conclusions

This study examined how underdog expectations influence employees’ interpersonal counterproductive work behavior (CWB-I) by developing a moderated mediation model grounded in self-determination theory. Specifically, we investigated two parallel psychological pathways—perceived insider status and moral disengagement—and examined the moderating role of organization-based self-esteem (OBSE).

The findings reveal that underdog expectations increase employees’ CWB-I through both relational and cognitive mechanisms. On the one hand, underdog expectations reduce perceived insider status, thereby weakening employees’ sense of organizational belonging and increasing interpersonal deviance. On the other hand, they foster moral disengagement, which enables employees to cognitively justify harmful interpersonal behaviors. Moreover, OBSE strengthens these indirect effects rather than buffering them, suggesting that individuals with high organizational self-worth may be more sensitive to negative evaluative cues.

Overall, this study advances understanding of how negative social expectations shape workplace deviance by highlighting the joint role of identity-based and moral cognitive processes under the framework of self-determination theory. It also underscores the complex role of self-concept in shaping employees’ behavioral responses to evaluative threats in organizations.

## Figures and Tables

**Table 1:** Means, Standard Deviations and Variable Correlations.

Variable	*M*	*SD*	1	2	3	4	5	6	7	8	9
1. Gender	1.39	0.49	-								
2. Age	2.23	0.93	0.05	-							
3. Education	1.53	0.75	0.09	−0.22 **	-						
4. Organizational Tenure	2.43	1.18	0.06	0.39 **	−0.20 **	-					
5. Underdog Expectations	2.40	0.76	−0.02	0.08	−0.11	0.08	(0.75)				
6. Perceived Insider Status	3.52	0.51	0.05	0.09	−0.07	0.09	−0.21 **	(0.72)			
7. Moral Disengagement	2.27	0.67	−0.16 *	−0.01	0.10	−0.14 *	0.30 **	−0.53 **	(0.91)		
8. Organization-Based Self-Esteem	3.32	0.63	−0.03	0.02	0.06	0.13	−0.17 **	0.66 **	−0.21 **	(0.91)	
9. Interpersonal Counterproductive Work Behavior	2.34	0.65	−0.09	−0.08	0.17	−0.11	0.17 *	−0.45 **	0.54 **	−0.21 **	(0.89)

Note. *n* = 221, *M* = Mean, *SD* = Standard Deviation. * *p* < 0.05; ** *p* < 0.01.

**Table 2:** Results of Confirmatory Factor Analysis.

Model	χ^2^	*df*	χ^2^/*df*	CFI	TLI	RMSEA	SRMR
5-factor model	182.59	80	2.28	0.95	0.93	0.08	0.06
4-factor model ^a^	264.91	84	3.15	0.91	0.89	0.10	0.10
3-factor model ^b^	423.14	87	4.86	0.83	0.80	0.13	0.13
2-factor model ^c^	665.53	89	7.48	0.71	0.66	0.17	0.14
1-factor model	1108.12	90	12.31	0.49	0.40	0.23	0.16

Note. *n* = 221. ^a^ 4-factor model: Perceived insider status and organization-based self-esteem combined to one factor; ^b^ 3-factor model: Perceived insider status, underdog expectations and organization-based self-esteem combined to one factor; ^c^ 2-factor model: Perceived insider status and organization-based self-esteem combined to one factor.

**Table 3:** Hierarchical Regression Analysis.

VAR	PIS				MD							CWB-I		
	M1	M2	M3	M4	M5	M6	M7	M8	M9	M10	M11	M12	M13	M14
Control Variables														
GEN	0.05	0.04	0.08	0.09	−0.16 *	−0.16 *	−0.17 **	−0.18 **	−0.10	−0.10	−0.08	−0.02	−0.08	−0.02
AGE	0.05	0.06	0.07	0.07	0.07	0.06	0.06	0.05	−0.02	−0.03	0.01	−0.06	0.00	−0.06
EDU	−0.06	−0.08	−0.13 *	−0.13 *	0.11	0.14 *	0.15 *	0.15 *	0.16 *	0.18 **	0.14 *	0.11	0.15 *	0.11
OT	0.06	0.07	−0.04	−0.05	−0.14	−0.15 *	−0.12	−0.11	−0.06	−0.07	−0.04	0.01	−0.04	0.01
Independent Variable														
UE		−0.23 **	−0.11 *	−0.10		0.32 ***	0.29 ***	0.27 ***		0.20 **			0.11	0.04
PIS											−0.43 ***		−0.41 ***	
MD												0.53 ***		0.51 ***
OBSE			0.65 ***	0.61 ***			−0.16 *	−0.10						
UE × OBSE				−0.12 *				0.20 **						
F	0.86	3.01 *	31.28 ***	28.13 ***	3.08 *	7.69 ***	7.66 ***	8.12 ***	2.52 *	3.91 **	12.56 ***	19.02 ***	11.07 ***	15.86 ***
R^2^	0.02	0.07 *	0.47 ***	0.48 ***	0.05 *	0.15 ***	0.18 ***	0.21 ***	0.05 *	0.08 **	0.23 ***	0.31 ***	0.24 ***	0.31 ***
ΔR^2^		0.05 **	0.45 ***	0.01 *		0.10 ***	0.12 ***	0.03 **		0.04 **	0.18 ***	0.26 ***	0.15 ***	0.2 ***

Note. *n* = 221, * *p* < 0.05; ** *p* < 0.01; *** *p* < 0.001. VAR = variable; UE = underdog expectations; PIS = perceived insider status; MD = moral disengagement; CWB-I = interpersonal counterproductive work behavior; OBSE = organization-based self-esteem; GEN = gender; EDU = education; OT = organizational tenure.

## Data Availability

The data presented in this study are available on reasonable request from the corresponding author due to privacy and ethical restrictions approved by the Institutional Review Board of Xiamen University.
